# REVIEW ON THE COMPLEX RELATION BETWEEN RETIREMENT AND COGNITION: UNRAVELING THE MIXED EVIDENCE

**DOI:** 10.1093/geroni/igz038.093

**Published:** 2019-11-08

**Authors:** Linn Elena Zulka, Isabelle Hansson, Linda B Hassing

**Affiliations:** 1 Department of Psychology, University of Gothenburg, Gothenburg, Sweden; 2 Department of Psychology, University of Gothenburg, Gothenburg, Vastra Gotaland, Sweden

## Abstract

A systematic review from 2017 revealed a great research gap concerning the question if retirement affects cognitive function. Since then, several longitudinal studies have been published, calling for an updated review. The aim of this review is to provide an update with a focus on different retirement operationalization, different cognitive outcomes, and potential mediators like occupational experiences. Twenty peer-reviewed studies with longitudinal designs were included. The results revealed no clear pattern regarding the association between retirement and the cognitive outcomes. Study results varied in relation to factors like occupational experiences, differences in study quality, and cognitive domains. To get an insight into mechanisms behind the relation between retirement and cognitive functioning, more complex study designs are needed that take into account the impact of pre-retirement factors, different retirement related aspects, and the varying effects depending on cognitive domain.

